# A diet rich in fermentable fiber promotes robust changes in the intestinal microbiota, mitigates intestinal permeability, and attenuates autoimmune uveitis

**DOI:** 10.1038/s41598-023-37062-8

**Published:** 2023-07-04

**Authors:** Yukiko K. Nakamura, Christina Metea, Victor Llorenç, Lisa Karstens, Ariel Balter, Phoebe Lin

**Affiliations:** 1 Casey Eye Institute, Oregon Health and Science University, Portland, OR USA; 2 Clinic Institute of Ophthalmology, Clinic Hospital of Barcelona, Barcelona, Spain; 3 Departments of Medical Informatics and Clinical Epidemiology, Oregon Health and Science University, Portland, OR USA; 4 Cole Eye Institute, Cleveland Clinic Foundation, Cleveland, OH USA

**Keywords:** Immunology, Eye diseases

## Abstract

Therapeutic approaches for noninfectious uveitis have expanded greatly over the past 10 years, but are limited by potential side effects and limited efficacy. Thus, therapeutic approaches that include less toxic, potentially preventative strategies to manage noninfectious uveitis are essential areas of study. Diets rich in fermentable fiber are potentially preventative in various conditions such as metabolic syndrome and type 1 diabetes. We studied the effects of various fermentable dietary fibers in an inducible model of experimental autoimmune uveitis (EAU) and found that they differentially modulated uveitis severity. A high pectin diet was the most protective, reducing clinical disease severity through the induction of regulatory T lymphocytes and the suppression of Th1 and Th17 lymphocytes at peak ocular inflammation in either intestinal or extra-intestinal lymphoid tissues. The high pectin diet also promoted intestinal homeostasis as shown by changes in intestinal morphology and gene expression, as well as intestinal permeability. Pectin-induced modulation of intestinal bacteria appeared to be associated with protective changes in immunophenotype in the intestinal tract, and correlated with reduced uveitis severity. In summary, our current findings support the potential for dietary intervention as a strategy to mitigate noninfectious uveitis severity.

## Introduction

Noninfectious uveitis, the most prevalent form of uveitis in developed countries, comprises a group of sight-threatening conditions^1,2^ for which effective treatment options are limited. Recent studies support the impact of an intestinal dysbiosis, intestinal barrier dysfunction, and the corresponding immune system dysregulation in the pathogenesis of various inflammatory diseases including autoimmune uveitis^3–6^. An intestinal dysbiosis can be driven by multiple external factors in a genetically susceptible host, such as low fiber diet, underlying comorbidities, antibiotic intake, or infection^4^.

Epidemiological studies throughout the world have shown that diets high in fiber reduce the incidence and mortality of many diseases including colon cancer, cardiovascular disease, infectious disease, diabetes, and obesity, whereas diets high in saturated fat have demonstrated opposite effects^7–10^. These beneficial effects of a high fiber diet occur through promotion of gut microbial production of short chain fatty acids (SCFAs), as well as through microbiota-independent effects on intestinal epithelium, both of which can promote intestinal and immune homeostasis^11^. We previously published that exogenous oral administration of SCFA (propionate) reduced the severity of uveitis in an experimental autoimmune uveitis (EAU) model partially through induction of regulatory T cells (Tregs) in the gut, prevention of effector T cell trafficking between the gut and the spleen during the course of EAU, and by reversing changes in intestinal morphological and gene expression characteristics brought on by the diseased state^12^. These findings were consistent with results from other studies administering SCFAs in various animal models of other immune-mediated diseases^13–18^. Our group also previously demonstrated that increased intestinal permeability occurred during the course of uveitis in EAU^3^, although it is unclear what role this disruption has in pathogenesis of ocular inflammation and whether or not therapeutic interventions that reduce uveitis severity work partially through reversing this effect.

In this work, we hypothesized that diets high in fermentable fibers that are more likely to promote endogenous intestinal bacterial production of SCFAs, would reduce uveitis severity through immune modulation and reduction in intestinal permeability in EAU.

## Results

### Fermentable dietary fibers modulate the severity of uveitis in EAU

To investigate whether high fiber diets influence EAU development and severity, the following six diets were administered ad libitum to C57Bl/6 J mice starting from day -35 (week -5) prior to immunization (immunization day = day 0) until euthanasia at 2 weeks post-immunization, which is the expected time point of peak intraocular inflammation in these mice: (1) diet without dietary fiber (NoFib); (2) standard rodent diet containing 5% non-fermentable insoluble cellulose (Chow); (3) diet containing 45% kcal fat (mainly consisting of saturated fat) and 5% non-fermentable insoluble cellulose (Fat); (4) diet containing 10% fermentable soluble pectin (Pectin); (5) diet containing 10% fermentable soluble inulin (Inulin); or 6) diet containing fermentable insoluble resistant starch-2-producing high amylose maize corn starch (Hylon^®^ VII), which is equivalent to 18% total dietary fiber (RS)^15^(Suppl. Table S1). These diets were chosen because the administration of fermentable dietary fibers is known to promote SCFA production by intestinal bacteria, whereas a high fat diet, akin to the western diet, has been shown to be pro-inflammatory^9,19–23^.

**Figure 1:**
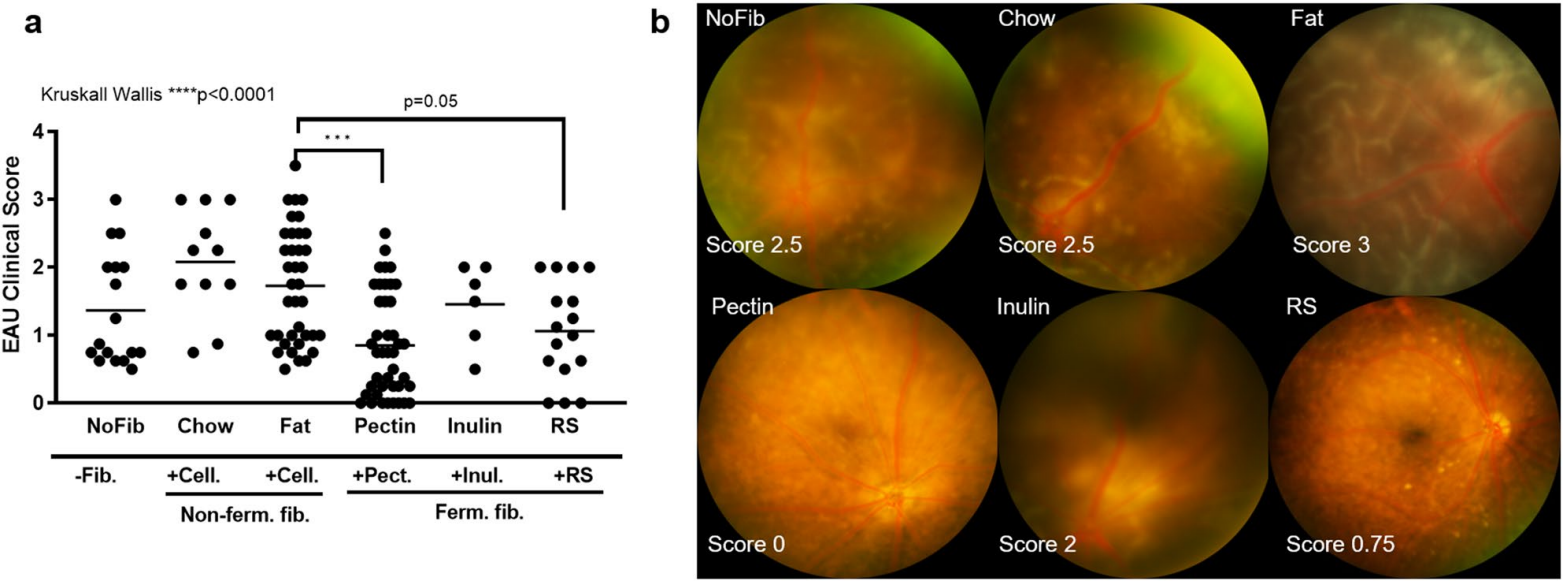
A diet high in fermentable dietary fiber attenuates uveitis in experimental autoimmune uveitis (EAU). (**a**) Krusall Wallis of uveitis clinical scores at 2 weeks post-immunization in various fermentable fiber rich diets (pectin, inulin, resistant starch) and control diets (no fiber, standard chow, high fat) in C57Bl/6 J EAU mice. (**b**) Fundus photograph images exhibiting differences in uveitis clinical score due to different diets. n = 6–42 animals/group; *p* < 0.001*** with [Fat] as the reference diet demonstrates the Bonferonni-corrected *p* value; means are shown in the graph; + Cell: cellulose-rich diets; Ferm. Fib: fermentable dietary fiber;

Diet significantly affected uveitis clinical score as shown by a Kruskall Wallis comparison (*p* < 0.0001) (Fig. 1a). Both the pectin and resistant starch diets significantly lowered EAU clinical scores compared to the high fat diet using a Mann-Whittney, whereas a diet high in inulin-type fiber had minimal effect on clinical score (Fig. 1a, b)(Clinical score means ± SD, p-values in the Mann–Whitney comparison: Pectin: 0.851 ± 0.757 vs. Fat: 1.726 ± 0.846, *p* < 0.0001; RS: 1.063 ± 0.733 vs. Fat *p* = 0.01), although it is possible that inulin and resistant starch diet groups were underpowered to show a difference. The pectin diet was noted to have the largest effect on reducing uveitis clinical score, and was significant after Bonferroni correction given that five comparisons were made (high fat diet used as reference group). The high fat diet did not appear to significantly worsen uveitis clinical score compared to regular chow on an individual Mann-Whittney (Fig. 1a).

Licholai et al.^24^ reported that a high fat diet given ad libitum results in overconsumption and consequent weight gain. To investigate the potential confounding effects of these factors on disease outcome, we looked at change in weight and food consumption among the different diets. None of the diets impacted body weight or food consumption except for the high fat diet. Female mice given the high fat diet had significant weight gain without increased food intake (female weight gain Fat: 133.3 ± 11.88% vs. Chow: 111.8 ± 3.24%, *p* = 0.008; vs. Pectin: 110.07 ± 4.97%, *p* = 0.0002) (Suppl Fig. 1a–c). In contrast, male mice given the high fat diet had increased weight gain with increased food consumption (Suppl. Fig. 1a–c)(male weight gain Fat: 151.9 ± 6.54% vs. Pectin: 134.3 ± 26.32%, *p* = 0.01; vs. Chow 138.5 ± 28.62%, n/s)(male food intake Fat: 2.893 ± 0.186 g/mouse/day vs. Pectin: 2.509 ± 0.185 g/mouse/day, *p* < 0.0001; vs. Chow: 2.527 ± 0.215 g/mouse/day, *p* < 0.0001). The discrepancy between male and female mice is not unexpected given that female mice have lower rates of hyperphagia than males fed a high fat diet, yet female mice still may develop morbid obesity from the high fat content.

### A high pectin diet alters intestinal morphology in EAU

Given that the high pectin diet had the largest effect on EAU clinical score, we focused further study on this diet compared to the high fat diet. We had previously demonstrated that exogenously administered oral SCFAs modulated various parameters of intestinal homeostasis such as intestinal morphology, gene expression, and permeability in EAU^12^ and sought to investigate a diet that increased endogenous production of SCFAs by the gut bacteria. The high pectin diet was selected because it has been shown to increase SCFA concentrations in the colon^20,25,26^. The high pectin diet induced increases in both acetate and propionate concentration in the intestinal tract (as shown by cecal stool concentrations), but decreased butyrate and valerate (acetate Pectin 2w: 321.6 ± 95.83 vs. Fat 2w: 282.2 ± 62.93, *p* = 0.05; propionate Pectin 2w: 105.5 ± 45.41 vs. Fat 2w 57.48 ± 16.88, *p* < 0.0001; butyrate Pectin 2w: 28.56 ± 12.25 vs. Fat 2w: 69.44 ± 15.63, *p* < 0.0001; valerate Pectin 2w: 6.29 ± 2.38 vs. Fat 2w: 12.61 ± 2.32, *p* < 0.0001)(Suppl. Fig. 2). Why the pectin diet reduces butyrate and valerate is unclear except that perhaps at 7 weeks post-initiation of diet, perhaps there are compensatory changes that result in reduction of some SCFAs, while maintaining a predominant SCFA like propionate, and to a lesser extent, acetate.

**Figure 2:**
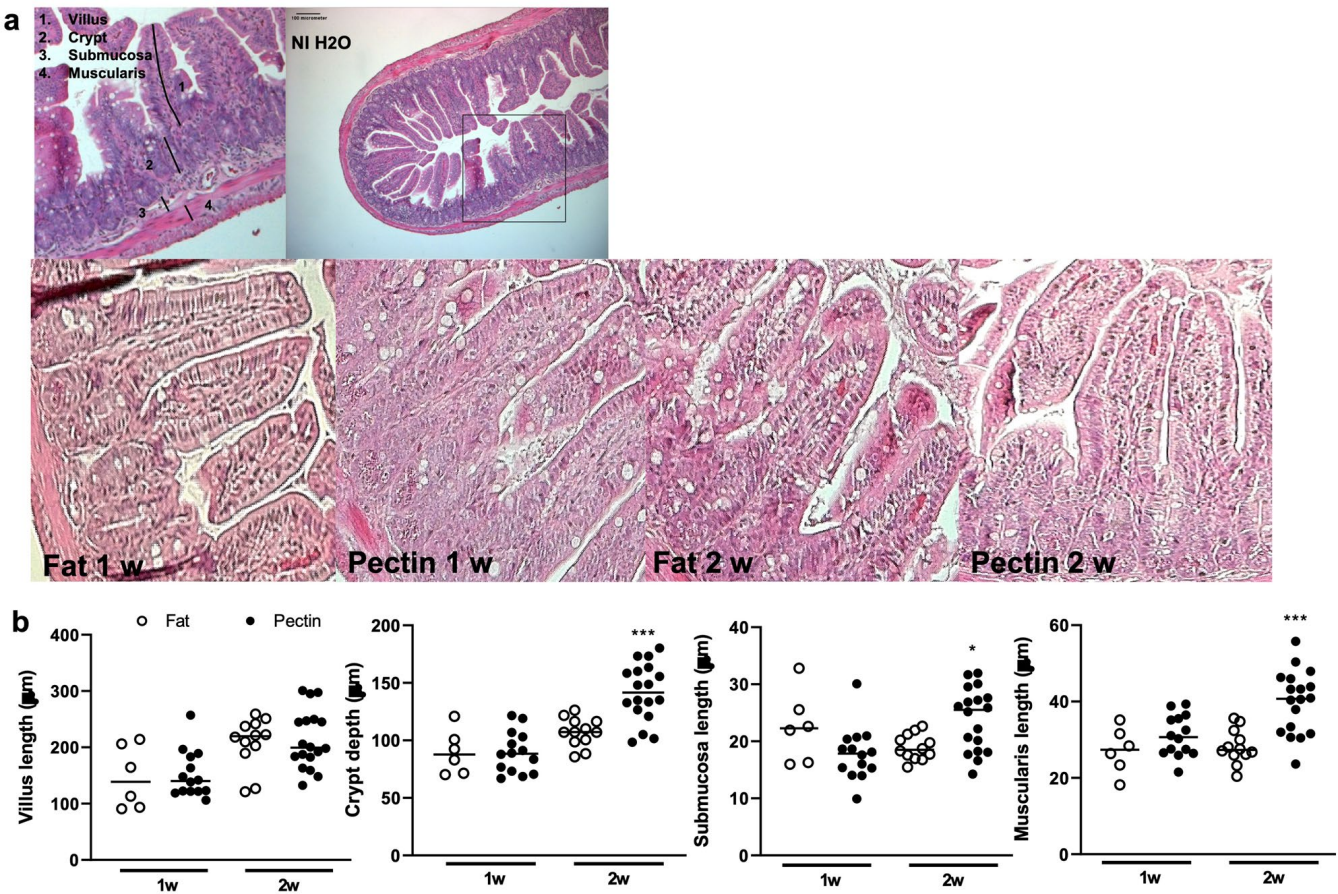
Changes in intestinal morphology in pectin versus high fat diet in EAU. (**a**) Representative hematoxylin and eosin-stained ileum sections from C57Bl/6 J non-immunized (NI) mouse illustrating sample measurements of villus, crypt, submucosa, and muscularis (Top panel), as well as high fat and pectin-fed EAU mice at 1 or 2 weeks post-immunization (bottom panels). (**b**) Quantification of ileal measurements in fat vs. pectin diets. 1w: 1 week; 2w: 2 weeks (post-immunization); n = 6–18 animals/group; *p* < 0.05*, *p* < 0.001***; means are shown in the graph.

Intestinal morphological changes were evaluated by measuring the thickness of the following ileal layers: the villus, crypt, submucosa, and muscularis. We had previously shown that temporary decreases in these various intestinal measures occurred during the course of untreated EAU at 1 week post-immunization^3^. At 2 weeks post-immunization in EAU animals, the pectin diet promoted elongation of the crypt, submucosa, and muscularis compared to the high fat diet (Fig. 2a, b)(crypt Pectin 2w: 141.6 ± 24.93 μm vs. Fat 2w: 107.2 ± 12.38 μm, *p* = 0.0004; submucosa Pectin 2w: 23.89 ± 5.52 μm vs. Fat 2w: 18.94 ± 2.23 μm, *p* = 0.02; muscularis Pectin 2w: 39.9 ± 8.27 μm vs. Fat 2w: 28.17 ± 4.44 μm, *p* = 0.0002). Our results appear to support the intestinal smooth muscle hypertrophy and increased intestinal crypt mass and cell counts reported in other studies with high fiber diets^27–29^. The pectin diet also seemed to stabilize the decreases in crypt depth, villus length, and muscularis length at 1 week post-immunization that occur during the course of EAU that we had found in prior studies^3^.

### A high pectin diet attenuates intestinal hyperpermeability and alters intestinal gene expression in EAU

**Figure 3:**
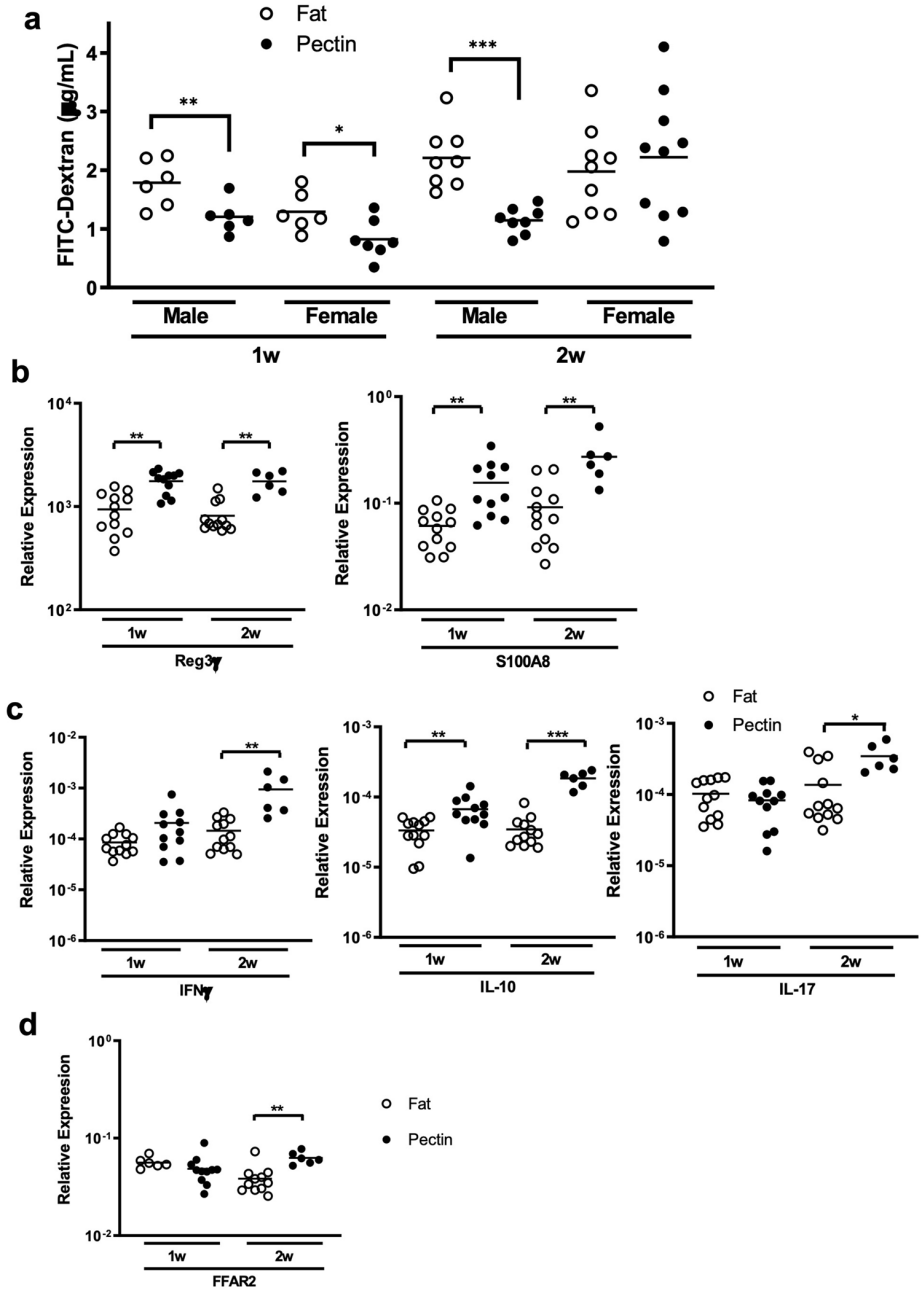
Pectin-induced changes in intestinal permeability and ileal gene expression. (**a**) Changes in intestinal permeability as measured by FITC-dextran assay in male and female pectin vs. high fat diet-fed EAU mice. (**b**) Differences in ileal anti-microbial peptide gene expression in combined male and female EAU mice. (**c**) Differences in ileal cytokine gene expression in EAU mice. (**d**) Pectin-induced increases in SCFA receptor FFAR2 transcript at 2 weeks post-immunization in EAU mice. n = 6–12 animals/group; *p* < 0.05*, *p* < 0.01**, *p* < 0.001***; means are shown in the graph.

To examine intestinal epithelial barrier function, we performed an in vivo intestinal permeability assay using oral gavage of FITC-dextran. We previously demonstrated increased intestinal permeability using this assay during the course of untreated EAU in a different mouse strain (B10RIII-H2r-H2)^3^. Compared to the high fat diet, the high pectin diet significantly reduced intestinal permeability measured by serum FITC-dextran levels during the first week post-immunization in both male and female EAU mice, as well as in male mice at 2 weeks, but had no effect in female mice at 2 weeks potentially due to gender-based differences in diet consumption (male Pectin 1w: 1.204 ± 0.277 μg/ml vs. Fat 1w: 1.787 ± 0.405 μg/ml, *p* = 0.009; male Pectin 2w: 1.147 ± 0.221 μg/ml vs. Fat 2w: 2.212 ± 0.522 μg/ml, *p* = 0.0002)(female Pectin 1w: 0.824 ± 0.333 μg/ml vs. Fat 1w: 1.293 ± 0.337 μg/ml, *p* = 0.04; female Pectin 2w: 2.223 ± 1.047 μg/ml vs. Fat 2w: 1.98 ± 0.738 μg/ml, n/s) (Fig. 3a and Suppl. Fig. 1).

Next, we examined ileal gene expression involved in host defense and inflammation, including anti-microbial peptides (AMPs) and cytokine production. The pectin diet significantly increased both Reg3γ and S100A8 transcript levels in EAU mice at both 1 and 2 weeks post-immunization (Reg3γ Pectin 1w: 1760 ± 428.9 vs. Fat 1w: 937.4 ± 400.1, *p* = 0.0005; Pectin 2w: 1756 ± 409.5 vs. Fat 2w: 813 ± 290.6, *p* = 0.0004) (S100A8 Pectin 1w: 0.1557 ± 0.0886 vs. Fat 1w: 0.06123 ± 0.02466, *p* = 0.0007; Pectin 2w: 0.2723 ± 0.1351 vs. Fat 2w: 0.09169 ± 0.06115 ± , *p* = 0.001) (Fig. 3b). This result was the reverse of the decrease in Reg3γ we had seen during untreated EAU in prior studies^3^. Also, the pectin diet significantly increased IL-10 as well as IFNγ and IL-17 cytokine transcript levels at 2 weeks post-immunization during peak uveitis (IL-10 Pectin 2w: 18 ± 4.63 × 10^–5^ vs. Fat 2w: 3.46 ± 1.83 × 10^–5^, *p* = 0.0001)(IFNγ Pectin 2w: 94 ± 74 × 10^–5^ vs. Fat 2w: 15 ± 9.78 × 10^–5^, *p* = 0.0004)(IL-17 Pectin 2w: 3.5 ± 1.6 × 10^–4^ vs. Fat 2w: 1.4 ± 1.3 × 10^–4^, *p* = 0.02) (Fig. 3c).

FFAR2/GPR43 is a SCFA receptor through which SCFAs regulate signaling pathways involved in various physiological events including host defense and metabolism^30,31^. The pectin diet upregulated the ileal transcript levels of FFAR2/GPR43, at peak ocular inflammation (Pectin 2w: 0.0628 ± 0.0092 vs. Fat 2w: 0.0383 ± 0.0130, *p* = 0.003) (Fig. 3d).

### A high pectin diet modulates T cell subsets

**Figure 4:**
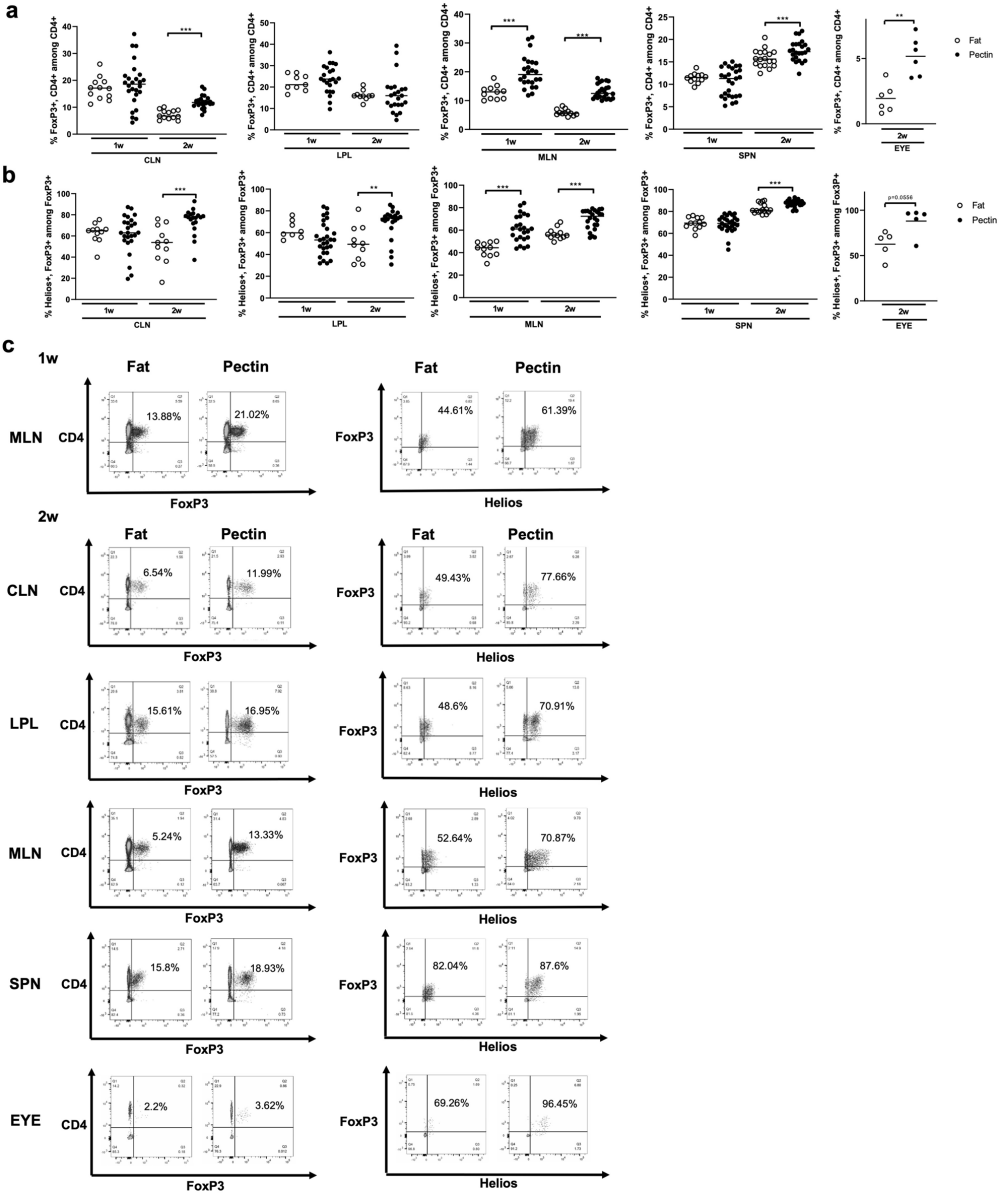
Pectin-induced differences in regulatory T cell prevalence in intestinal and extraintestinal lymphoid tissues. Flow cytometric quantitation of (**a**) FoxP3+, CD4+ T cells and (**b**) Helios+, FoxP3+, CD4+ T cells, in pectin versus high fat-diet fed EAU mice. (**c**) Example flow cytometry plots from which prevalence calculations were derived (percentages in bold are FoxP3+, CD4+ cells/total CD4+ cells) CLN: cervical lymph node; LPL: lamina propria lymphocytes; MLN: mesenteric lymph node; SPN: spleen. n = 5–27 animals/group; *p* < 0.05*, *p* < 0.01**, *p* < 0.001***; means are shown in the graph.

We have shown previously that administration of exogenous SCFAs promoted regulatory T cells (Tregs) in EAU^12^. We performed flow cytometry to assess the frequency of CD4+ Tregs, characterized by expression of FoxP3 and/or with co-expression of the Helios marker given that Helios expression is known to enhance the suppressive function of CD4+ Tregs^32^. We examined Treg frequency in pectin vs. fat-fed EAU mice in the following intestinal and extra-intestinal lymphoid tissues: cecal + colonic lamina propria lymphocytes (LPL); the cervical lymph nodes (CLN); the mesenteric lymph nodes (MLN); the spleen (SPN); and the eye (EYE). At 1 week post-immunization, prior to uveitis onset, we observed higher frequencies of FoxP3+, CD4+ Tregs in the MLN in pectin-fed EAU mice compared with high fat diet (FoxP3 MLN Pectin 1w: 19.85 ± 5.51% vs. Fat 1w: 12.86 ± 2.4%, *p* < 0.0001), whereas at 2 weeks (peak EAU), there were increases in Treg frequency in all extra-intestinal tissues evaluated, including the eye (Fig. 4a, c)(FoxP3 CLN Pectin 2w: 11.94 ± 2.33% vs. Fat 2w: 7.31 ± 1.72%, *p* < 0.0001; MLN Pectin 2w: 13.14 ± 2.53% vs. Fat 2w: 5.9 ± 1.1%, *p* < 0.0001; SPN Pectin 2w: 17.49 ± 2.29% vs. Fat 2w: 15.8 ± 1.94%, *p* = 0.02; EYE Pectin 2w: 5.17 ± 1.48% vs. Fat 2w: 1.95 ± 1.09%, *p* = 0.009). At 1 week, the pectin diet increased the frequency of Helios + , FoxP3+, CD4+ Tregs in the MLN (Helios MLN Pectin 1w 61.3 ± 11.81% vs. Fat 1w: 43.02 ± 6.30%, *p* < 0.0001), whereas by 2 weeks Helios + Tregs were increased in all tissues including in the intestinal LPL (Fig. 4b, c)(Helios CLN Pectin: 2w 74.11 ± 12.34% vs. Fat 2w: 52.49 ± 17.81%, *p* = 0.0004; MLN Pectin 2w: 69.57 ± 8.64% vs. Fat 2w: 56.76 ± 4.9%, *p* = 0.0002; SPN Pectin 2w: 87.45 ± 3.22% vs. Fat 2w: 82.4 ± 4.35%, *p* = 0.0009; LPL Pectin 2w: 68.37 ± 14.79% vs. Fat 2w: 50.43 ± 16.21%, *p* = 0.008; EYE Pectin 2w: 88.03 ± 15.56% vs. Fat 2w: 62.52 ± 14.74%, *p* = 0.06).

**Figure 5:**
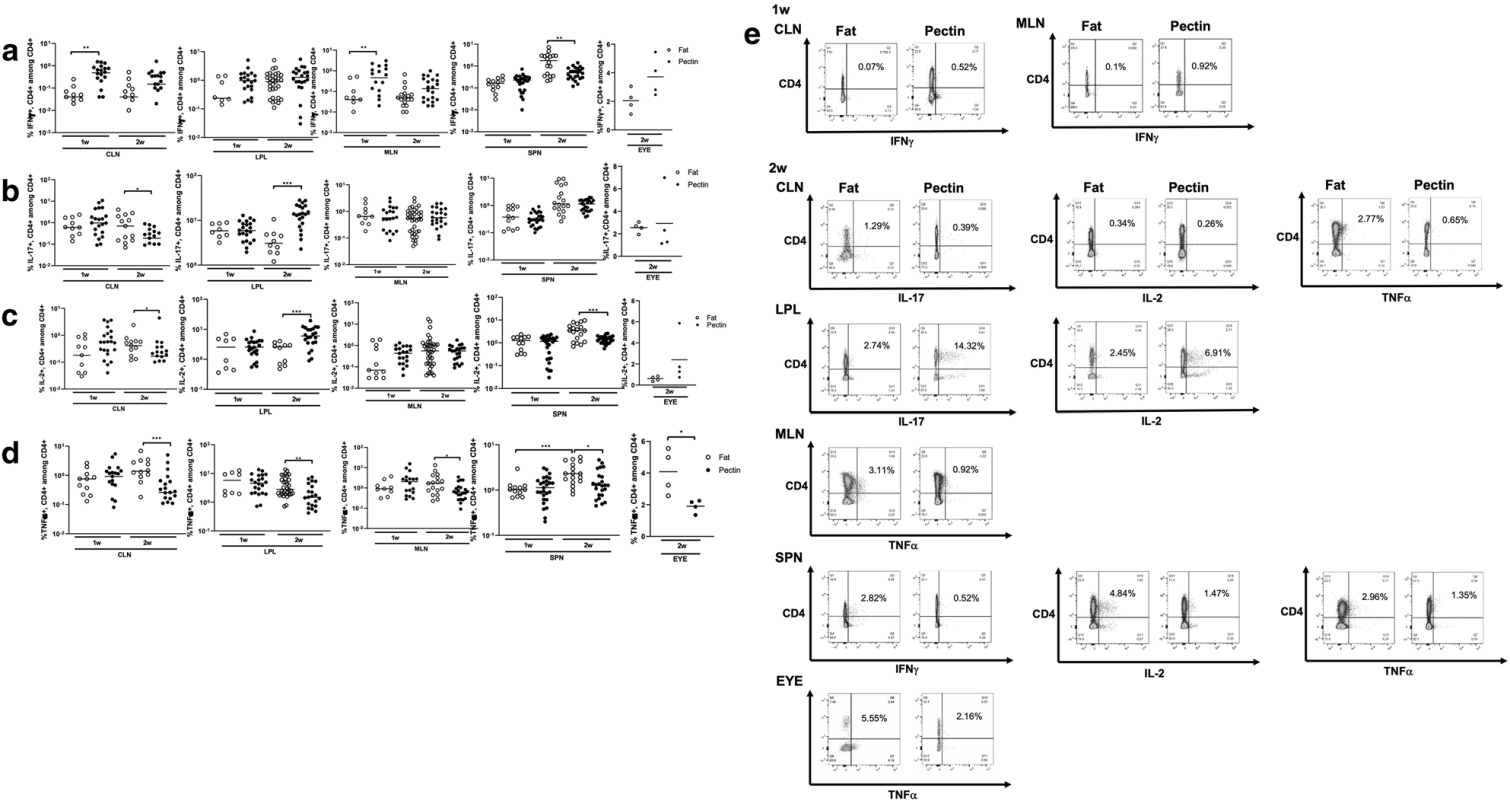
Pectin-induced effector T cell prevalence in intestinal and extra-intestinal lymphoid tissues. Flow cytometric quantitation of (**a**) IFNγ+, CD4+ (Th1); (**b**) IL-17+, CD4+ (Th17); (**c**) IL-2+, CD4+ ; and (**d**) TNFα+ , CD4+ ; T cells in pectin vs. high fat-diet fed EAU mice; (**e**) Example flow cytometry plots from which prevalence calculations were derived in (**a**)–(**c**); CLN: cervical lymph node; LPL: lamina propria lymphocytes; MLN: mesenteric lymph node; SPN: spleen. n = 4–27 animals/group; *p* < 0.05*, *p* < 0.01**, *p* < 0.001***; means are shown in the graph.

Effector T cells were also investigated. The pectin diet reduced the frequency of Th1 (IFNγ+, CD4+) cells in the spleen and Th17 (IL-17+, CD4+) cells in CLN at 2 weeks post-immunization (Fig. 5a, b, e)(IFNγ SPN Pectin 2w: 0.538 ± 0.323% vs. Fat 2w: 2.07 ± 1.87%, *p* = 0.004)(IL-17 CLN Pectin 2w: 0.262 ± 0.277% vs. Fat 2w: 1.182 ± 1.281%, *p* = 0.02). IL-2-producing CD4+ T cells were reduced in CLN and spleen, whereas TNFα-producing, CD4+ T cells were reduced in all the extra-intestinal tissues examined including the eye, at 2 weeks post-immunization (Fig. 5c, d, e)(IL-2 CLN Pectin 2w: 0.365 ± 0.917% vs. Fat 2w: 0.53 ± 0.604%, *p* = 0.02; SPN Pectin 2w: 0.584 ± 0.427% vs. Fat 2w: 2.99 ± 4.98%, *p* = 0.007)(TNFα CLN Pectin 2w: 0.683 ± 1.17% vs. Fat 2w: 2.12 ± 1.82%, *p* = 0.0008; MLN Pectin 2w: 0.935 ± 0.895% vs. Fat 2w:2.63 ± 3.32%, *p* = 0.04; SPN Pectin 2w: 1.62 ± 1.162% vs. Fat 2w: 2.6 ± 1.422%, *p* = 0.01; EYE Pectin 2w:1.908 ± 0.406% vs. Fat 2w: 4.08 ± 1.415%, *p* = 0.03).

Thus, the pectin diet appears to ameliorate uveitis, in part, through T lymphocyte subset modulation. These findings support our previous study demonstrating that oral administration of SCFA attenuated uveitis by inducing Tregs and suppressing Th1 and Th17^12^.

### A high pectin diet promotes intestinal microbial changes associated with protection from severe EAU

**Figure 6:**
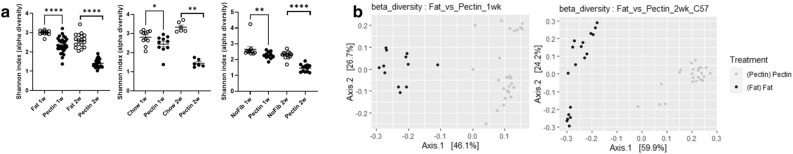
Alpha and beta diversity of the intestinal microbiome in diet-altered EAU mice. (**a**) Shannon index alpha diversity at 1 (1w) and 2 weeks (2w) after immunization in high Fat-, regular Chow-, No Fiber-, vs. pectin-fed EAU mice. (**b**) Weighted unifrac beta diversity principle coordinates analysis plots at 1 and 2 weeks after immunization in pectin versus fat-fed EAU mice. n = 5–18 animals/group; *p* < 0.05*, *p* < 0.01**, *p* < 0.0001****; means are shown in the graph.

To identify, classify, and quantify the intestinal commensal bacteria, we utilized 16 s rRNA gene sequencing in diet-fed EAU mice. Alpha diversity, a measure of species richness and evenness, was significantly lower in mice on the pectin diet than the high fat, standard (Chow) diet, and the no fiber (NoFib) diets with all alpha diversity measures analyzed (Shannon Diversity indices shown in Fig. 6a at both 1 and 2 weeks after immunization. This result across all diet comparisons suggests that this alpha diversity-lowering effect is due to the pectin diet rather than an increased alpha diversity due to the control diets. Beta diversity, specifically, weighted UniFrac, also showed significant differences in the pectin diet vs. the high fat diet at both 1 and 2 weeks post-immunization, with good separation along Axis 1 on the PCoA plot (Fig. 6b)(1w permanova *p* = 0.001; 2w *p* = 0.001) and pectin diet vs. other control diets as well (supplemental Fig. 3).

**Figure 7:**
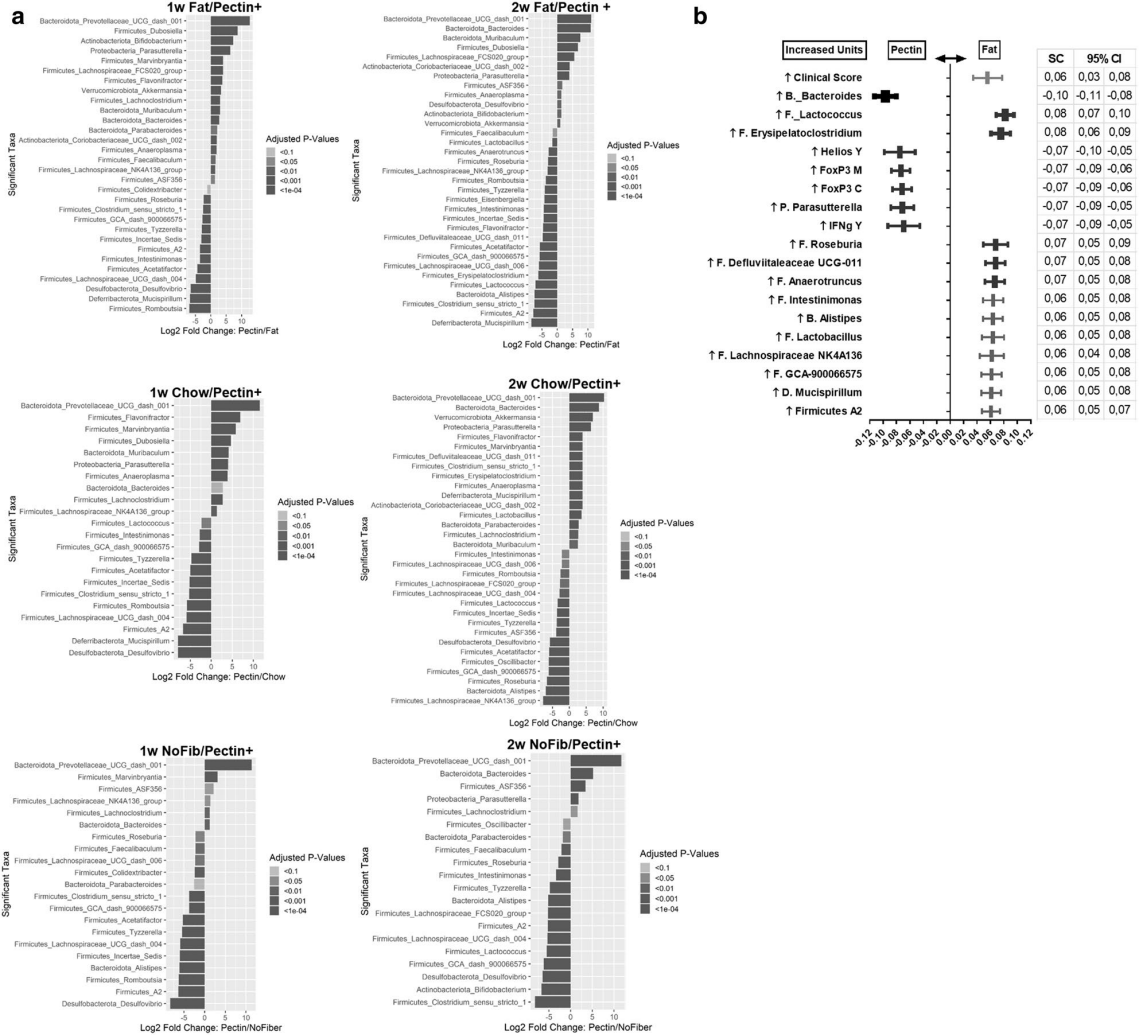
Differential abundance of bacterial genera and integrative statistical analysis in different diet-fed EAU mice. (**a**) Deseq2 plots showing differential abundance as log2fold change in pectin vs. control diets (Fat, regular Chow, No Fiber); positive log2fold changes are those that are increased in pectin compared to the control diet at 1 (1w) and 2 (2w) weeks post-immunization. (**b**) Partial least squares-discriminant analysis of the pectin vs. high fat diets at 2 weeks post-immunization; SC: standardized coefficient; CI: confidence interval; n = 5–18 animals/group; for (**a**), adjusted *p* values are shown as grayscale intensity in the bars (scale shown), with darker intensity/black demonstrating lower *p* values.

By DESeq2 analysis, multiple bacteria were differentially abundant in the pectin vs. high fat EAU mice at 1 week and 2 weeks post-EAU induction (Fig. 7a, top panel). The pectin diet increased abundance of *Parasutterella*, *Bacteroides*, *Bifidobacterium* and *Akkermansia* at 1 and/or 2 weeks post-immunization compared with more than one control diet (Chow, NoFib, Fat), suggesting that these changes are pectin-induced enhancements rather than depletions due to the control diets (Fig. 7a, top, middle, and lower panels). Alternatively, the pectin diet depleted *Desulfovibrio*, *Roseburia*, *Lachnospiraceae NK4A136*, *Tyzzerella, Romboutsia*, and *Mucispirillum* among other bacteria, compared to more than 1 diet at 1 or 2 weeks post-immunization, suggesting that these changes are pectin-induced depletions rather than control diet-induced enhancements of these bacteria.

To determine association between pectin-induced intestinal bacterial modulation and the immunophenotype of various tissue sites in EAU animals, a partial least squares discriminant analysis, which combines a principal components analysis and logistic regression (PLS-DA), between pectin and fat diets was performed utilizing the intestinal bacterial genera relative abundance and the prevalence of T effectors and Tregs from different tissue sites at 2 weeks post-immunization (peak uveitis). Clinical score was used as an internal marker (expected to have significant correlation), and standardized coefficients (SC) equal to or greater than that with the clinical score, with either a positive or negative sign (correlation), were shown (Fig. 7b). Given that animals did not get significant intraocular inflammation at 1 week post-immunization, only the 2 week time point was utilized for the PLS-DA. Using the above approach, the variables that were identified accounted for approximately 90% of the discriminatory power between these diets in the data set. On PLS-DA, covariates independently associated with the pectin diet included an increased abundance of *Bacteroides* and *Parasutterella*, which co-associated with increased prevalence of Tregs in the MLN and CLN as well as increased prevalence of Helios + Tregs in the eyes (Fig. 7b). The correlation matrix revealed that *Bacteroides* significantly correlated with Tregs in the CLN (Spearman r = 0.526, 95%CI 0.127–0.778; *p* = 0.009). PLS-DA also showed increased abundance of many (12) bacterial genera in fat-fed mice, mostly *Firmicutes*, including *Lachnospiraceae NK4A136* and *Defluviitalaceae UCG-011*, which co-associated with depletion in Tregs in the above-mentioned tissues. Both of the latter bacteria correlated significantly with higher uveitis clinical scores on the correlation matrix (*Lachnospiraceae NK4A136* vs. Clinical score: Spearman r = 0.589, 95%CI 0.127–0.841; *p* = 0.012, *Defluviitalaceae UCG-011* vs. Clinical score: Spearman r = 0.560**,** 95%CI 0.088 to 0.826; *p* = 0.017).

## Discussion

EAU is a primarily T-cell mediated mouse model of uveitis similar to various forms of human uveitis. It can be induced by subcutaneous injection of an emulsion containing a retinal antigen, interphotoreceptor retinoid binding protein (IRBP) peptide, along with an adjuvant, typically killed mycobacteria. Classically, ocular inflammation occurs after a priming or “licensing” phase in the spleen during the first 2–3 days, during which a few uveitogenic T cells develop the capability to invade the target tissue (the eye). Peak ocular inflammation does not usually occur until mostly non-autoreactive effector cells are recruited to the eye *en masse* at 2–3 weeks post-induction. Caspi and colleagues have shown by adoptive transfer and neutralizing antibody studies that autoreactive Th1 and Th17 CD4+ T cells mediate EAU, depending on the EAU model utilized^33^. Different microbial adjuvants result in predominance of Th1 vs. Th17 cells in EAU^34^, with mycobacterial adjuvant causing a predominantly Th17 response, while the bacterial endotoxin, lipopolysaccharide, promotes a Th1-predominant effect. However, more recently, Horai et al.^35^ discovered that activation of autoreactive retina-specific Th17 cells occurs within the intestinal tract by a commensal gut bacterial antigen, although the cognate intestinal bacterial antigen remains to be identified. Taken together, one can hypothesize that, depending on which intestinal microbiota are present, either Th1 or Th17 cells promote uveitis, and that the microbiota can potentially educate autoreactive Th17 cells in the gut that are directly pathogenic for the eye.

By contrast, Tregs, characterized by expression of the transcription factor, FoxP3, play a crucial role in resolution of inflammation, as their depletion after resolution of uveitis results in relapse^36^. Pre-existing, likely thymic, Tregs raise the threshold for uveitis susceptibility of disease, since their depletion enhances IRBP-induced uveitis. In uveitis patients, studies have shown decreased Treg prevalence during active uveitis, increased Tregs in remission, and decreased Tregs in association with more severe uveitis^37–41^. A vast and growing role of intestinal Tregs, above and beyond Tregs found in other tissues such as the spleen and peripheral lymph nodes, has more recently been demonstrated. For instance, colonic RORγt + Tregs induced by intestinal bacteria are more suppressive than other Tregs in a T-cell mediated model of colitis^42^. Korn and colleagues showed that microbiota-derived factors are not just important for inducing peripheral Tregs in the gut, but also for maintaining gut-resident thymic Tregs in the intestinal lamina propria^43^. Despite this breadth of mechanistic data in the literature, very little is known about how intestinal immunity, integrally affected by diet and intestinal gut bacterial alterations, affect ocular immunity.

In this study, we have made several novel findings. First, we demonstrated that the administration of a diet high in the fermentable fiber pectin ameliorated uveitis through the induction of regulatory T lymphocytes and suppression of effector T lymphocytes in various lymphoid tissues. Although most types of human uveitis do not mimic EAU exactly, they have in common that Th1 and Th17 cells are large players in many types of human uveitis, and that it is plausible that exposure to different environmental adjuvants from the gut bacteria or elsewhere can potentially promote an aberrant immune response in the eye. The induction of Tregs in the gut by the pectin diet are potentially the most important finding in this study given the previously demonstrated importance of Tregs in inducing remission in uveitis patients. Second, we observed that intestinal morphology, gene expression, and intestinal permeability were altered by the pectin diet. Specifically, the high pectin diet decreased intestinal permeability compared to the high fat diet. In prior studies, we had demonstrated that an increase in intestinal permeability is associated with EAU even prior to onset of intraocular inflammation^3^. The pectin diet appears to reverse this trend in both male and female mice. The intestinal crypt, which is considered a more immunologically active site at the base of the intestinal villi, was decreased during the course of EAU in prior studies^3^, but was increased in this study by the pectin diet. While at first glance there seems to be discrepancy between the pectin-induced elevation in IL-10 in the iluem, which is consistent with the enhancement of Tregs in the gut, and the increases in IL-17 and IFN-gamma, which are typically inflammatory mediators in uveitis and other conditions, one must remember that IL-17 can serve a protective role in repairing the intestinal mucosa after inflammation, and is consistent with increases in antimicrobial peptides that might be beneficial. This contradictory role of IL-17 in the gut versus elsewhere in the body is evident by the finding that IL-17 inhibition promotes IBD in patients^44^.

Third, we found that the abundance of certain intestinal bacteria that were modulated by the pectin diet seemed to be associated with the proportion of intestinal T lymphocyte subtypes that can be important in re-establishing intestinal as well as systemic/ocular immune homeostasis. Specifically, *Bifidobacterium*, which is known to produce short chain fatty acids that can induce Treg differentiation in the gut^45^, and *Akkermansia*, whose protective effects on the gut mucosa may stabilize or reduce intestinal permeability^46^, were both increased in abundance by the pectin diet in EAU mice at both time points (1 and 2 weeks), potentially explaining why this dietary intervention increased Tregs and reversed intestinal permeability changes associated with the EAU model. Pectin also depleted *Desulfovibrio*, which we had shown previously to be strongly associated with the uveitic state in EAU^6^. Whether or not the association between *Parasutterella* and Tregs in various tissues in protection against EAU represented a true Treg-inducing action of these bacteria, or was an association due to a compensatory phenomenon secondary to other intestinal bacterial changes induced by pectin, remains to be seen. For instance, *Tyzzerella* is depleted in response to increasing amounts of *Bifidobacterium* in the gut, and its depletion is thought to be beneficial^47^. On the other hand, *Mucispirillum,* depleted by the pectin diet in our study, has been associated with inflammatory bowel disease^48^. The strong association between a high pectin diet and increased *Bacteroides* is consistent with previous reports comparing obese and obesity-related diseased populations and healthy controls^49^. Moreover, *Bacteroides fragilis* and its component polysaccharide A, in particular, has been demonstrated to be a pivotal driver of Treg differentiation and suppression, able to abolish intestinal inflammation when given to germ-free experimental models of inflammatory bowel disease^50^. Pectin indeed increased *Bacteroides*, although 16S sequencing could not provide species level data to confirm this as *Bacteroides fragilis*^50^. Thus, overall, we have shown beneficial gut bacterial changes induced by the pectin diet that can promote intestinal immune homeostasis, potentially help induce remission in uveitis patients through induction of Tregs, and stabilize intestinal barrier function, the latter of which has been shown in other diseases to be important in re-establishing/maintaining a healthy state.

Diet- and fiber type-dependent uveitis attenuation can be explained by different intestinal microbial compositions. Dietary fibers have exhibited different physiological effects, as well as chemical properties. As for their chemical properties, pectin, inulin, and types of resistant starch are water soluble and fermentable fibers, whereas cellulose (contained in the standard chow and high fat diets) is water insoluble and non-fermentable. Fermentation patterns depend on the molecular weight, chain length, and structure of the fiber. SCFAs, fermentation products derived from dietary fibers, can promote the growth of probiotics, such as *Bifidobacteria* and *Lactobacilli*, by lowering colonic pH^51^. With regard to their physiological effects, resistant starch and bifidogenic inulin can produce butyrate, whereas pectin induces the production of acetate, propionate, and butyrate through the promotion of *Lactobacillus* and *Bifidobacterium* in various animal and culture models^14,15,51–53^. Certain strains of *Akkermansia*, *Propionibacterium*, and *Faecalibacterium* are considered possible candidate probiotics, in addition to strains of *Lactobacillus* and *Bifidobacterium*, which have been intensively studied and clinically used as probiotics^54^. When exogenous SCFAs, propionate and acetate, are given in mice, they are more potent inducers of Treg differentiation than butyrate^55^, which might explain why despite the reduction in butyrate production over time with the pectin diet, there was such a potent effect on Tregs in our study.

An additional possible mechanism by which the pectin diet ameliorated uveitis is the modulation of T lymphocyte profiles through the SFCA-activated FFAR/GPR43 pathway. The intestinal microbiota regulates host immunity and metabolism partly by modulating the production of SCFAs that serve as FFAR/GPR43 agonists^55,57–61^. In our current study, the differential microbial abundances coincided with pectin-induced production of propionate (and to a lesser extent, acetate) and FFAR2/GPR43 expression at peak ocular inflammation. These findings may support the idea of the role of SCFAs in upregulating Tregs through FFAR2/GPR43 activation, though further research is required including abrogation of this effect in FFAR2 -/- mice or using FFAR2 antagonism.

The strengths of this study include the finding of a potentially effective and novel therapeutic adjuvant to treat autoimmune uveitis using dietary intervention, of which there is surprisingly little in the published literature, as well as the putative mechanism of this efficacy demonstrated to occur through enhancement of intestinal Tregs and improvement of intestinal permeability and histology. The weaknesses of this study include the limited study numbers for multiple diets (such as with the inulin and resistant start diets) that might limit a true comparison of the various diets for the most effective diet, the fact that this study was performed in mice rather than humans and may not translate to efficacy in uveitis patients, and that the macronutrient composition varies between diet groups and may impact interpretation of the findings. Another limitation was that gender specific differences were investigated only in terms of dietary intake and intestinal permeability but were not powered per gender to look at immunophenotypic differences that might occur. Also, use of 16 s sequencing data does not resolve to the strain level nor tell us what aspect of the bacterial genera influenced by the pectin diet are promoting the protective effect in uveitis. Many of these limitations are being actively studied in our lab.

In conclusion, we report novel findings demonstrating diet-induced alterations of intestinal microbial and T lymphocyte profiles, thus promoting intestinal and immune homeostasis. Administration of fermentable dietary fiber may be a potentially non-toxic adjunctive to other immunomodulatory treatments for noninfectious uveitis.

## Materials and methods

### Ethics statement

The experimental protocols on mice were approved by the Oregon Health & Science University (OHSU) Institutional Animal Care and Use Committee (IACUC) (Protocol ID IP00000374) with strict ethical and humane treatment guidelines according to Association for Research in Vision and Ophthalmology Statement for the Use of Animals in Ophthalmic and Visual Research. The study is reported in accordance with ARRIVE guidelines.

### Animals (C57BL/6J, Kaede/C57BL/6J) and diets

C57BL/6J mice were purchased from the Jackson Laboratory (Sacramento, CA, USA). In accordance with the institutional policies for animal health and well-being at OHSU, mice were maintained in ventilated cages under HEPA-filtered barrier conditions and were fed an assigned diet starting five weeks prior to immunization until termination. The diets we tested were: (1) diet without fiber (No Fiber: D14071801N, Research Diets, Inc., New Brunswick NJ, USA); (2) standard rodent diet (Chow: D10012G, Research Diets, Inc.); (3) diet containing 45% kcal fat (Fat: D19052101, Research Diets, Inc.; TD.08811, ENVIGO, Madison, WI, USA); (4) diet containing 10% Pectin (Pectin: D18062704, Research Diets, Inc.); (5) diet containing 10% Inulin (Inulin: D18062703, Research Diets, Inc.); and (6) diet containing resistant starch (RS2)-producing high amylose maize corn starch (RS: TD.120455 with Hylon-VII^®^, ENVIGO). All diets were gamma-irradiated and were given ad libitum. Food intake and body weight of individual animals in each group were recorded weekly. Isoflurane mask anesthesia was used on mice during examination and immunization procedures in methods approved by the IACUC committee. Animals were sacrificed at 1 or 2 week time points after EAU induction (see below) utilizing a two-step process with CO2 exposure, followed by cervical dislocation, in accordance with the approved IACUC protocol.

### EAU induction

Male and female 8- to 12-week-old C57Bl/6J mice were immunized subcutaneously into the base of the tail and each thigh with 200 µl total of the emulsion containing IRBP651–670 (300–500 µg peptide/animal; GenScript, Piscataway, NJ, USA), incomplete Freund’s adjuvant (IFA; Sigma-Aldrich, St. Louis, MO, USA), and heat-inactivated *Mycobacterium tuberculosis* antigen (5 mg/ml IFA; BD, Franklin Lakes, NJ, USA). An additional adjuvant, pertussis toxin from *Bordetella pertussis* (1 µg dose/animal; Sigma-Aldrich), was injected subcutaneously once at the time of immunization per previously published work^6^. Clinical EAU scores (a grading scale of 0–4) were assessed by fundus examination using a 90D lens (Volk, Mentor, OH, USA) and indirect ophthalmoscope (Keeler, Sacramento, CA, USA) weekly until termination^62^. Fundus images were taken with Micron IV (Phoenix Technology Group, Pleasanton, CA, USA). When possible, the examiner was blinded to treatment group by use of a secondary technician creating a randomized cage order and covering the treatment group listed on the cage. The same examiner was used for all studies.

### Cell isolation

Single cell suspensions of the following lymphoid tissues were obtained by processing through a 70 µm cell strainer: the spleen (SPN; through RBC removal with ammonium-chloride-potassium [ACK] lysis buffer), the cervical lymph node (CLN), and the mesenteric lymph node (MLN). Lamina propria lymphocytes (LPL) were isolated from the cecum and colon. Intestinal contents were removed beforehand. The cecum and colon were cut into 1 cm-long pieces and were digested in RPMI media with 5 mM EDTA (37 °C, 30 min × 2) and subsequently with 0.1 mg/ml collagenase II (Sigma-Aldrich) and 0.1 mg/ml DNase I (Roche, San Francisco, CA, USA) at 37 °C for 45 min twice (total 90 min). LPL were isolated after resuspending digested intestinal tissues in 30% Percoll (GE Healthcare, Pittsburgh, PA, USA) and then centrifuging the tissues layered on a 40%/60% Percoll gradient at 740 × g for 20 min. Two eyes from one animal were pooled. Enucleated eyes were minced after lens removal. The minced eye pieces were digested with 1 mg/ml collagenase D (Roche) and 15 µg/ml DNase I (Roche) at 37 °C for 40 min and processed through a 70 µm cell strainer.

### Flow cytometry

Flow cytometry was performed as previously reported^12^, using the same set of antibodies and reagents. For regulatory T cell (Treg) frequency assessment, single cell suspensions processed were stained with an anti-mouse CD4-FITC antibody and Live/Dead dye-efluor780 (eBioScience, San Diego, CA, USA) after Fc blocking with rat anti-mouse CD16/CD32 Fc block (BD Pharmingen, San Jose, CA, USA). The cell suspensions were fixed with the FoxP3 Staining Buffer Kit (eBioScience) overnight and subsequently stained with antibodies against intracellular proteins, FoxP3-APC (clone FJK-16 s, eBioScience) and Helios-efluor450 (clone 22F6, eBioScience). For Th1 and Th17 cell detection, single cell suspensions were stimulated with 500 ng/ml phorbal 12-myristate 13-acetate (PMA, Sigma-Aldrich) and 2 μg/ml ionomycin (Sigma-Aldrich) in RPMI media (10% FBS, penicillin/streptomycin, β-mercaptoethanol) containing GolgiStop/monensin (BD BioSciences) at 37 °C for 3 h. The stimulated cells were stained with an anti-mouse CD4-PE-Cy7 (clone GK1.5, BD BioSciences) antibody and Live/Dead dye-efluor780 and then fixed with intracellular cytokine detection kit (BD BioSciences) prior to staining with anti-mouse antibodies: IFNγ-FITC (clone XMG1.2, BD BioSciences), IL-17A-Alexa Fluor 647 (clone TC11-18H10, BD Pharmingen), IL-2-Alexa Fluor 700 (clone JES6-5H4, BD Pharmingen) and TNFα-PE (clone TN3-19.12), eBioScience). Flow cytometric data were obtained using an LSR Fortessa Cell Analyzer (BD BioSciences), and the data analyzed using FlowJo software (FlowJo LLC, Ashland, OR, USA). Flow cytometric data points were excluded if flow plot quality was poor for a given sample or if parent cell counts were not above a certain minimal threshold for each tissue type.

### Ileum structure measurement

After euthanasia, fresh ileum was collected and cut into approximately 7 mm-long pieces. Ileal fragments were fixed with 10% neutral buffered formalin (NBF) overnight, and then stored in 70% ethanol at 4 °C until tissue processing. The tissue was embedded in paraffin after tissue processing (Shandon Citadel 2000, Thermo Scientific) in the sagittal orientation, then sectioned transversely at 6 μm thickness, followed by hematoxylin–eosin staining. H&E-stained ileal tissue was photographed under the Leica DM 5000B microscope (Leica Microsystems Buffalo Grove, IL, USA). ImageJ was used to measure the thickness of the ileal villus, crypt, submucosa, and muscularis, in a process that was previously described^3^.

### Ileal gene expression

Fresh ileal tissue (7 mm-long fragment) was snap-frozen with liquid nitrogen, and stored at − 80 °C until RNA isolation. Total RNA was extracted with TRIzol (Thermo Fisher Scientific), and cDNA was synthesized at 37 °C using a cDNA synthesis kit (Thermo, Applied Biosystems). The same sets of primers and reagents were used in this study as described previously^12^. Gene expression of anti-microbial peptides (AMPs) was assessed with the following primer sets: Reg3γ (Mm00441128_g1), S100A8 (Mm00496696_g1), FFAR2/Gpr43 (Mm01176528_g1), and HPRT (Mm01545399_m1) as the housekeeping gene, using the Maxima Probe/ROX qPCR Master Mix (Thermo Fisher Scientific). Gene expression of various cytokines was quantified with custom-made primer sets: IFNγ(forward: 5′-GCG TCA TTG AAT CAC ACC TG-3′, reverse: 5′-TGA GCT CAT TGA ATG CTT GG-3′), IL-10 (forward: 5′-GGT TGC CAA GCC TTA TCG GA-3′, reverse: 5′-ACC TGC TCC ACT GCC TTG CT-3′), IL-17A (forward: 5′-CTC AAA GCT CAG CGT GTC CAA ACA-3′, reverse: 5′-TAT CAG GGT CTT CAT TGC GGT GGA-3′) and GAPDH (forward: 5′-TCA ACA GCA ACT CCC ACT CTT CCA-3′, reverse: 5′-ACC CTG TTG CTG TAG CCG TAT TCA-3′), as the housekeeping gene, using SYBR Green reagents (Quanta Biosciences, Beverly, MA, USA) performed on a ViiA 7 Real-time PCR machine (Thermo Fisher Scientific). Relative expression of these target genes to internal controls (HPRT or GAPDH) was calculated.

### In vivo intestinal permeability assay

An in vivo intestinal permeability assay was performed to examine intestinal barrier function. After a 4 h fast (food and water), mice were given FITC-labeled dextran (360 mg/kg body weight, MW4000; Sigma-Aldrich Corp.) via oral gavage. Whole blood was collected through cardiac puncture at 70 min post-gavage, and was centrifuged at 2500× *g* for 10 min to obtain serum. Serum was diluted with PBS pH 7.4 (1:6 vol/vol). Standard curves were obtained by serial dilution of FITC-dextran in nontreated serum diluted with PBS (1:6 vol/vol). Fluorescence intensity was measured (excitation 485 nm, emission 535 nm; SpectraMax iD3 Multi-Mode Microplate Reader, Molecular Devices, San Jose, CA, USA). FITC-dextran concentrations were calculated based on the standard curves.

### Short chain fatty acid analysis

Fecal sample extracts were prepared as described by Weitkunat and colleagues^22^. Following extraction and saponification, the samples were frozen and then lyophilized overnight using a Labconco FreeZone 2.5 Plus lyophilizer with a setting of 0.25 mBar (Labconco, Kansas City, MO, USA). The dry pellets were then dissolved by adding 50 μl of 5 M formic acid, 200 μl of acetone and then vortexed for 1 min with tubes capped tightly. After centrifugation at 15,000 rpm for 5 min at 4 °C, the top portion was transferred to an autosampler vial with insert and capped tightly. SCFA were separated using an Agilent 7890B GC with a 5977A MSD detector with an autosampler and split/splitless injector operated in split mode (Agilent Technologies, Santa Clara, CA, USA). The column was an Agilent HP-20-M capillary column (25 m, 0.32 mm id, 0.3 μm film thickness). Helium was the carrier gas at a flow rate of 2 ml/min. The injection port and auxiliary heater were maintained at 200 °C. A 1 μl sample was injected in split mode (1:10) at an initial oven temperature of 75 °C, held for 1 min and then increased at 10 °C/min to 140 °C, held for 8 min followed by 70 °C/min to 180 °C, held for 3 min and then returned to 75 °C. The mass spectrometer was operated at a source temperature of 220 °C and a MS quad temperature of 150 °C in positive electron impact mode. Fatty acids were detected by selected ion monitoring of ions at *m/*z 55, 57, 60, 73, 83, and 87, each with a 50 ms dell time after a solvent delay of 4 min. Peaks were identified by co-chromatography with standards from a Supelco Mix WSFA-2 (Sigma-Aldrich), and by comparison of the mass spectrum with the standard spectra in the NIST library by obtaining full scan spectra of each peak. Retention times for the analytes were: acetic acid, 5.0 min; proprionic acid, 6.0 min; butyric acid, 7.1 min; isovaleric acid, 7.6 min; valeric acid, 8.4 min; and the internal standard, isobutyric acid, 6.4 min. The instrument was controlled and data acquired using 5977 enhanced MassHunter version B.07.04.2260 and analyzed using Agilent MassHunter quantitative analysis, version B.07.0. Standard curves were generated from area ratios of authentic standards and the internal standard, isobutyric acid, and used for quantification of unknowns.

### 16S rRNA gene sequence processing, taxonomic classification, and diversity analyses

Cecal content was obtained using sterile equipment under a flow laminar hood immediately after termination and were stored at − 20 °C until microbial DNA isolation. Microbial DNA was isolated from frozen cecal contents with a DNA isolation kit according to protocol (Mo Bio Laboratories, Inc., Carlsbad, CA, USA). Amplification of the 16S small subunit rRNA gene was performed using standard protocols of the Earth Microbiome Project (www.earthmicrobiome.org)^63^. The V4 region of the 16S gene was targeted with universal primers 515 F/806RB and sequenced with an Illumina MiSeq. Standard methods to find unique phylogenetic units in the sequences based on the 16S rRNA locus were used; specifically, the Divisive Amplicon Denoising Algorithm (DADA) implemented in the DADA2 R package. The DADA algorithm denoises and demultiplexes 16S sequences to resolve distinct 16S sequences called amplicon sequence variants (ASVs). Microbial phylogenetic identification was performed by aligning the ASVs to the SILVA v.132 16S rRNA database (https://www.arb-silva.de/). The alpha diversity measure of within-sample community diversity (for both ASV and genera) was calculated using the VEGAN package in R. The beta-diversity measure of between-sample community diversity was calculated using weighted UNIFRAC (phyloseq package in R^64,65^. The UNIFRAC distances were also used to test for significant differences between two dietary interventions using the PERMANOVA test implemented in the adonis function in the VEGAN package using the default setting of 1000 permutations. To identify differentially abundant taxa between groups, we utilized Deseq2 analysis after quality filtering with a relative abundance cutoff of 0.2% and a prevalence cutoff of 10%. Deseq2 detects features with significant differential expression based on generalized linear models following a negative binomial distribution as we have done previously^6,66^.

### Statistical analysis

A nonparametric Mann–Whitney U test (with or without Bonferroni correction), Kruskal–Wallis, and D'Agostino-Pearson normality test were performed to compare groups, using GraphPad Prism 8 (GraphPad Software, Inc., La Jolla, CA, USA). Integrative statistics were calculated using a partial least squares, discriminant analysis (PLS-DA) among the diet-treated EAU mice including the relative abundance of bacteria at a genus level and the Treg and T effector prevalence calculated by flow cytometry analysis. A standardized coefficient (SC) greater than or equal to that achieved by clinical score at 2 weeks post-EAU induction, was utilized as the cutoff for the covariables that entered the model. Spearman’s correlation analysis was also used to look at correlations within specific diet groups (pectin and fat). Means are shown in graphs and *p* < 0.05 was generally considered statistically significant.

## Supplementary Information

Supplementary Information.

## Data Availability

The datasets generated and/or analyzed during the current study are available in the “DietMicrobiomeData” repository found at the following link: https://drive.google.com/drive/folders/1QqliU7WILWcKIC0nVgz7LGGe4chdMVD2?usp=sharing. The 16S sequencing data will be publicly available via the NCBI repository (an INSDC member repository) via the following persistent link, with BioProject ID: PRJNA884872 https://urldefense.com/v3/http://www.ncbi.nlm.nih.gov/bioproject/884872;!!Mi0JBg!JCwVibLyCB_fJfJbtd0Op8zAQGUDdylsnZUCAKnCSETpreQNYNaukLXKKtXFOs9WvRhUdrK3B2KPObC6XOHBZ5l-Y1F_d_eS$
