# Supplementary material for: A diet rich in fermentable fiber promotes robust changes in the intestinal microbiota, mitigates intestinal permeability, and attenuates autoimmune uveitis
Source: Sci Rep. 2023 Jul 4;13:10806. doi: 10.1038/s41598-023-37062-8 (PMC10319740; doi:10.1038/s41598-023-37062-8)
Supplement: Supplementary file 1 — Supplementary Information. [file 41598_2023_37062_MOESM1_ESM.pdf]

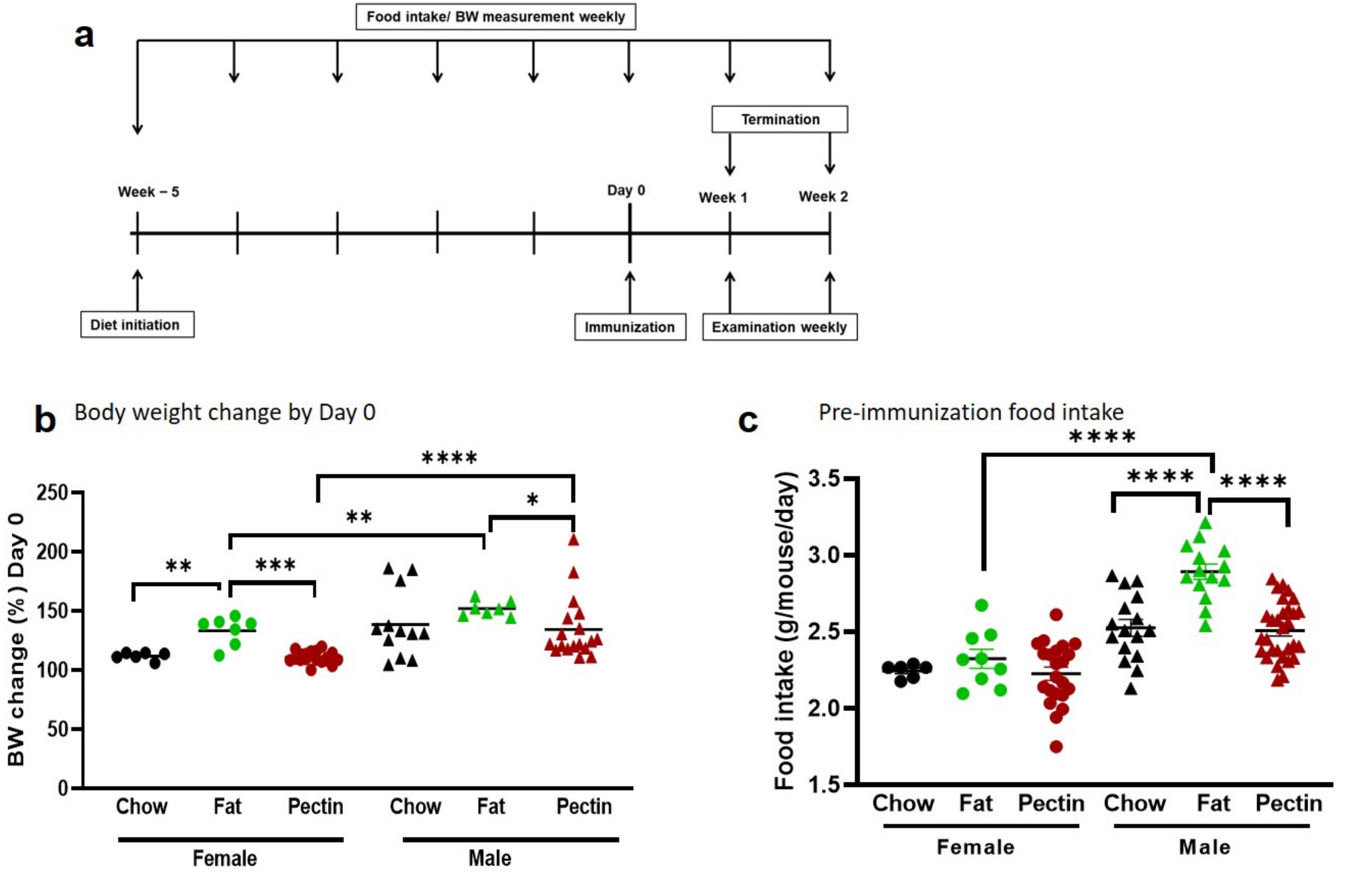

Supplemental Figure 1. (A) Study design and timing of dietary intervention. (B,C) Percentage body weight (BW) change and amount of food intake by day 0 (day of immunization prior to immunization) with different diets, regular Chow, high Fat diet, and pectin diet (means shown). n=6-18 animals/group; p<0.05*, p<0.01**, p<0.001***means are shown in graphs.

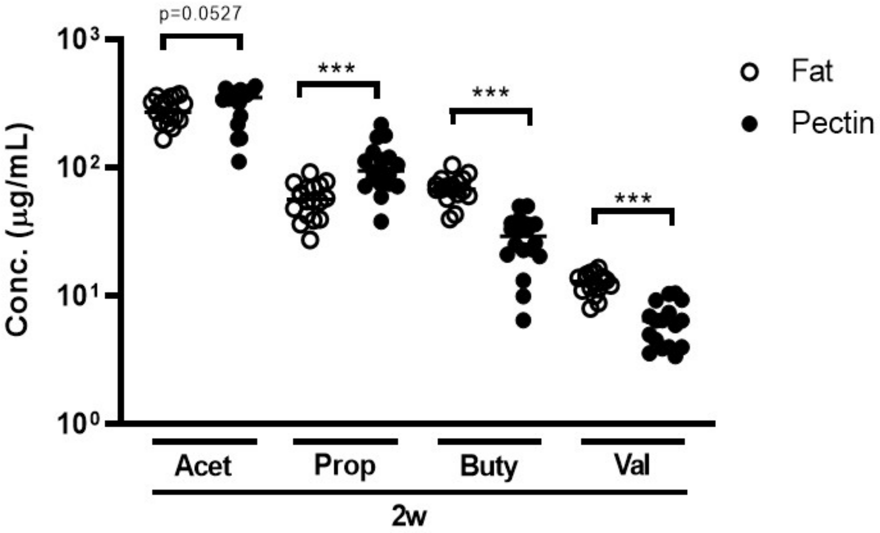

Supplemental Figure 2. Quantification of cecal short chain fatty acid (SCFA) content by GC-MS. n= 17-18 animals/group; p<0.001***; means are shown in the graph. Acet: acetate; Prop: propionate; Buty: butyrate; Val: valerate; 2w: 2 weeks post-immunization

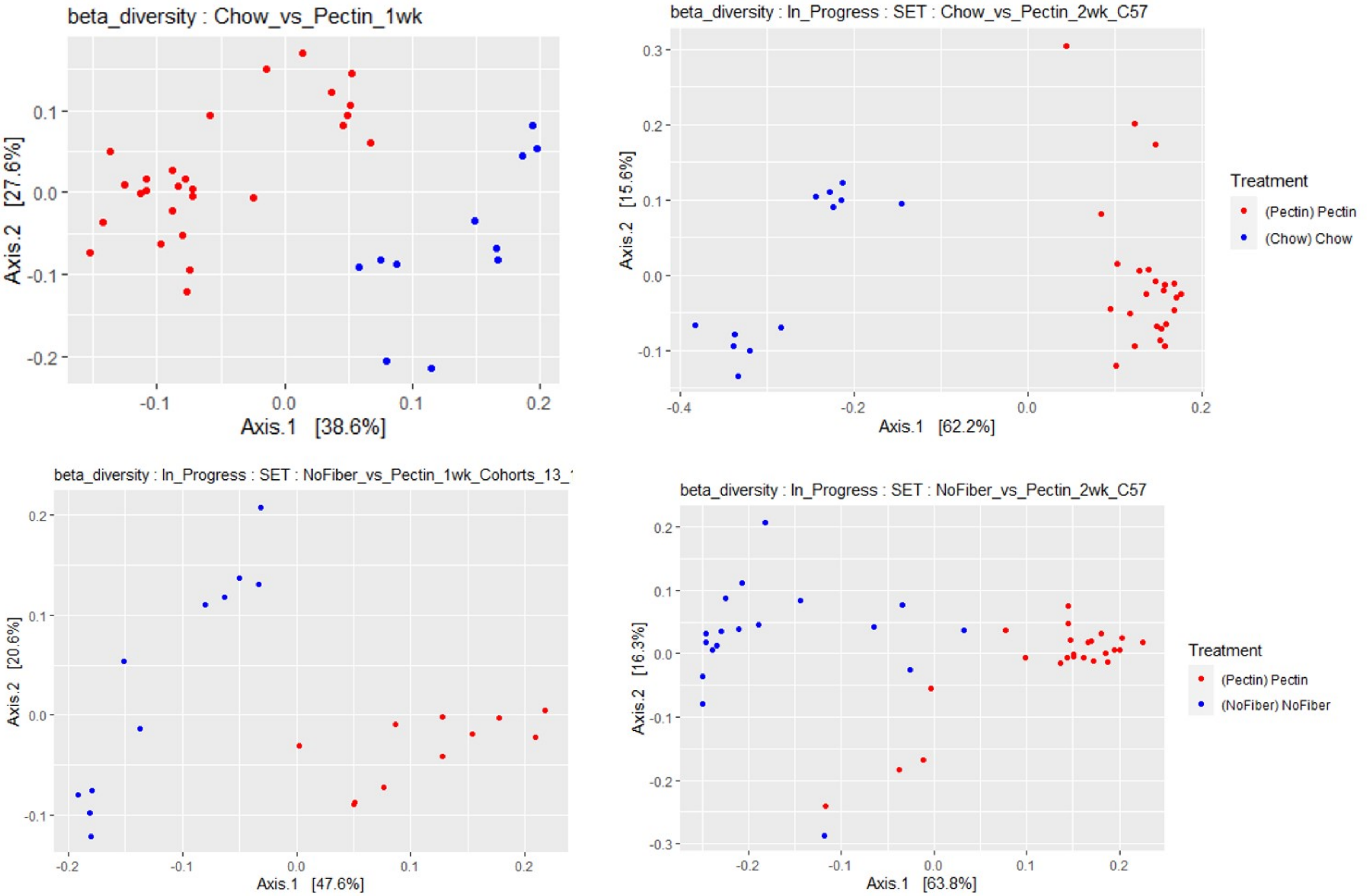

Supplemental Figure 3. Beta diversity (weighted unifrac) in Chow vs. Pectin and No Fiber vs. Pectin groups at 1 and 2 weeks post-immunization

Diet Type	No Fiber		Chow		Fat		Pectin		Inulin		Resistant Starch	
	gram%	kcal%	gram%	kcal%	gram%	kcal%	gram%	kcal%	gram%	kcal%	gram%	kcal%
Protein	21	20	20	20	20	17	20	20	20	20	18.6	22.3
Carbohydrate	67	64	64	64	48	40	59	59	69	64	50.6	60.9
Fat	7	16	7	16	23	43	7	16	7	16	6.2	16.8
Total				100		100		95		100		
kcal/gram	4.2	100	4		4.8		4		3.9		3.3	
Ingredient	gram	kcal	gram	kcal	gram	kcal	gram	kcal	gram	kcal	gram	kcal
Casein	200	800	200	800	195	780	200	800	200	800	210	840
L-Cystine	3	12	3	12	3	12	3	12	3	12	3	12
Corn Starch	397.486	1590	397.486	1590	56.86	227	347.5	1390	359.5	1438	0	0
High Amylose Corn Starch (Hylon-VII®)*	0	0	0	0	0	0	0	0	0	0	500	2000
Maltodextrin 10	132	528	132	528	60	240	132	528	132	528	100	400
Sucrose	100	400	100	400	340	1360	100	400	100	400	39.14	156.56
Cellulose, BW200	0	0	50	0	50	0	0	0	0	0	35	0
Resistant Starch (RS2)*	0	0	0	0	0	0	0	0	0	0	240	0
Inulin (1.5 kcal/gram)	0	0	0	0	0	0	0	0	101.3	152	0	0
Pectin (2 kcal/gram)	0	0	0	0	0	0	100	200	0	0	0	0
Soybean Oil	70	630	70	630	20	180	70	630	70	630	20	180
Milk Fat (or Lard)	0	0	0	0	210	1890	0	0	0	0	40	360
t-Butylhydroquinone	0.014	0	0.014	0	0.04	0	0.014	0	0.014	0	0.01	0
Mineral Mix S10022G	35	0	35	0	43	0	35	0	35	0	35	0
Vitamin Mix V10037	10	40	10	40	19	76	10	40	10	40	15	60
Choline Bitartrate	2.5	0	2.5	0	3	0	2.5	0	2.5	0	2.75	0
Total	950	4000	1000	4000	999.9	4765	1000.014	4000	1013.314	4000	1000	4008
Fiber (%)	0		5		4.7		10		10		18	

Supplemental Table 1. Diet contents
